# Responses of sorghum to cold stress: A review focused on molecular breeding

**DOI:** 10.3389/fpls.2023.1124335

**Published:** 2023-02-23

**Authors:** Pedro Fernando Vera Hernández, Leopoldo Ernesto Mendoza Onofre, Flor de Fátima Rosas Cárdenas

**Affiliations:** ^1^ Instituto Politécnico Nacional, Centro de Investigación en Biotecnología Aplicada, Ex-Hacienda San Juan Molino Carretera Estatal Tecuexcomac-Tepetitla, Tlaxcala, Mexico; ^2^ Colegio de Postgraduados, Programa de Producción de Semillas, Texcoco, Mexico

**Keywords:** sorghum, chilling, molecular breeding, genes, cold tolerance

## Abstract

Climate change has led to the search for strategies to acclimatize plants to various abiotic stressors to ensure the production and quality of crops of commercial interest. Sorghum is the fifth most important cereal crop, providing several uses including human food, animal feed, bioenergy, or industrial applications. The crop has an excellent adaptation potential to different types of abiotic stresses, such as drought, high salinity, and high temperatures. However, it is susceptible to low temperatures compared with other monocotyledonous species. Here, we have reviewed and discussed some of the research results and advances that focused on the physiological, metabolic, and molecular mechanisms that determine sorghum cold tolerance to improve our understanding of the nature of such trait. Questions and opportunities for a comprehensive approach to clarify sorghum cold tolerance or susceptibility are also discussed.

## Introduction

Plants are sessile organisms that are continuously confronted with a variety of abiotic stresses (Kidokoro et al., 2021; Rawat et al., 2021; Li et al., 2022, Kidokoro et al., 2022; Huang et al., 2022). Cold is a limiting factor that has been considered among the main abiotic stresses that interfere with plant growth and development (Achard et al., 2008; Mehrotra et al., 2020). Cold temperatures affect critical cellular functions such as photosynthesis, respiration, and membrane permeability (Rawat et al., 2021; Daems et al., 2022; Li et al., 2022). Meanwhile, the process of cold acclimation involves many physiological, biochemical, metabolic, and molecular changes (Achard et al., 2008; Bu et al., 2021; Yu et al., 2022; Rani et al., 2021; Goswami et al., 2022; Hu et al., 2022; Liu et al., 2022; Zhang et al., 2022).

Therefore, understanding the cold stress response is crucial for improving crops against cold (Adhikari et al., 2022; Liu et al., 2022). Molecular, cellular, and physiological mechanisms activated to respond to cold stress determine the plant’s susceptibility or tolerance to the environment (Goswami et al., 2022; Liu et al., 2022; Zhang et al., 2022). Several studies have examined the cold response in different plants and tissues (Zhou et al., 2021; Adhikari et al., 2022; Huang et al., 2022; Khanthavong et al., 2022; (Kidokoro et al., 2022; Zhang et al., 2022). In this sense, sorghum has been identified as a susceptible crop at low temperatures (Burow et al., 2011; Chopra et al., 2015). Sorghum is a cold-sensitive crop, but some varieties are cold tolerant (Leon-Velasco et al., 2009; Mansour et al., 2021). Adverse effects in sorghum such as reduction in germination, plant growth, and development are evident by cold stress (Ercoli et al., 2004; Chinnusamy et al., 2007; Janmohammadi et al., 2015; Ortiz et al., 2017; Laza et al., 2022). Integration of signal perception and transduction, gene expression, and molecules produced in response to cold stress is essential to understand sorghum tolerance and acclimatization (Mehrotra et al., 2020). The purpose of this paper is to review and discuss some of the research results that focused on the physiological and gene regulation as well as signal transductions that determine sorghum cold tolerance to improve the understanding of the nature of such trait.

## Cold tolerance: A rare trait in sorghum

Cold is one of the main factors that determine the distribution of agricultural species worldwide, limiting their productivity and yield (Mehrotra et al., 2020). It is challenging to determine when and where the sorghum domestication occurred. However, it is assumed that it occurred in Northeastern Africa (Morris et al., 2013) under hot and dry field conditions (Jain et al., 2010; Laza et al., 2022). Sorghum has been well adapted to drought and high-temperature environments where the growth of other cereals crop may be challenged. However, sorghum is a susceptible crop at low temperatures compared with most other cereals (Jain et al., 2010; Chopra et al., 2015). Some varieties of sorghum have been developed in the cooler highland areas of China and some parts of Africa (Knoll et al., 2008; Maulana et al., 2017). These sorghums are cold tolerant and can be adapted and grown in highlands where rainfall is scarce and erratic or the growing season is usually less suitable for other sorghum varieties (Leon-Velasco et al., 2009). It is a crop adapted to warm areas (Vera-Hernández et al., 2018), where minimum average temperatures during the growing period are generally maintained above 18°C (Reshma et al., 2019). The gradual expansion to high-altitude regions has led to the development of genotypes that are adapted to cold climates. Its introduction in other regions of the world has resulted in more cold-tolerant varieties, as well as early maturation and insensitive photoperiod cultivars that are able to grow under these adverse conditions.

The term “cold tolerance or chilling tolerance” is used to describe the ability of sorghum to germinate, grow, and produce seed under conditions of low temperature but above freezing temperatures (Singh, 1985; Osuna-Ortega et al., 2003; Vera-Hernández et al., 2018; Emendack et al., 2021). Low temperature is one of the main abiotic stressors that affect plant growth and development and is a principal determinant of the geographical distribution of plants (Hurry et al., 2002; Mehrotra et al., 2020). The cold temperatures restrict sorghum production by reducing the window periods for cultivation in locations where the crop is already grown and by spatially limiting its growth in colder regions. A few varieties of sorghum have been developed in the cooler highland areas and can be adapted and grown in highlands where rainfall is scarce and erratic. The growing season is usually less suitable for other sorghum varieties (Leon-Velasco et al., 2009).

Higher survival under cold conditions (Bekele et al., 2014; Parra-Londono et al., 2018) as well as early vigor and germination in cold environments (Burow et al., 2011; Fiedler et al., 2012; Parra-Londono et al., 2018) have been determined in sorghum plants with tolerance to cold stress. Likewise, high chlorophyll content (Fiedler et al., 2014), enhanced transpiration, and affected stomatal conductance (Ortiz et al., 2017) under cold conditions have been identified in sorghum. Furthermore, an increase in anthocyanin levels (Chopra et al., 2017) has been suggested to have a protective function in chilling exposure (Krol et al., 1995). Other molecules, including genes and lipids, have been shown to change in response to cold stress (Marla et al., 2017). The effect of cold temperature on sorghum will depend on the stress intensity and duration, the developmental stage of the crop at the time at which low temperatures prevail (Emendack et al., 2021), and the variety of sorghum.

## Adverse effects of cold stress exposure in sorghum

**Figure 1:**
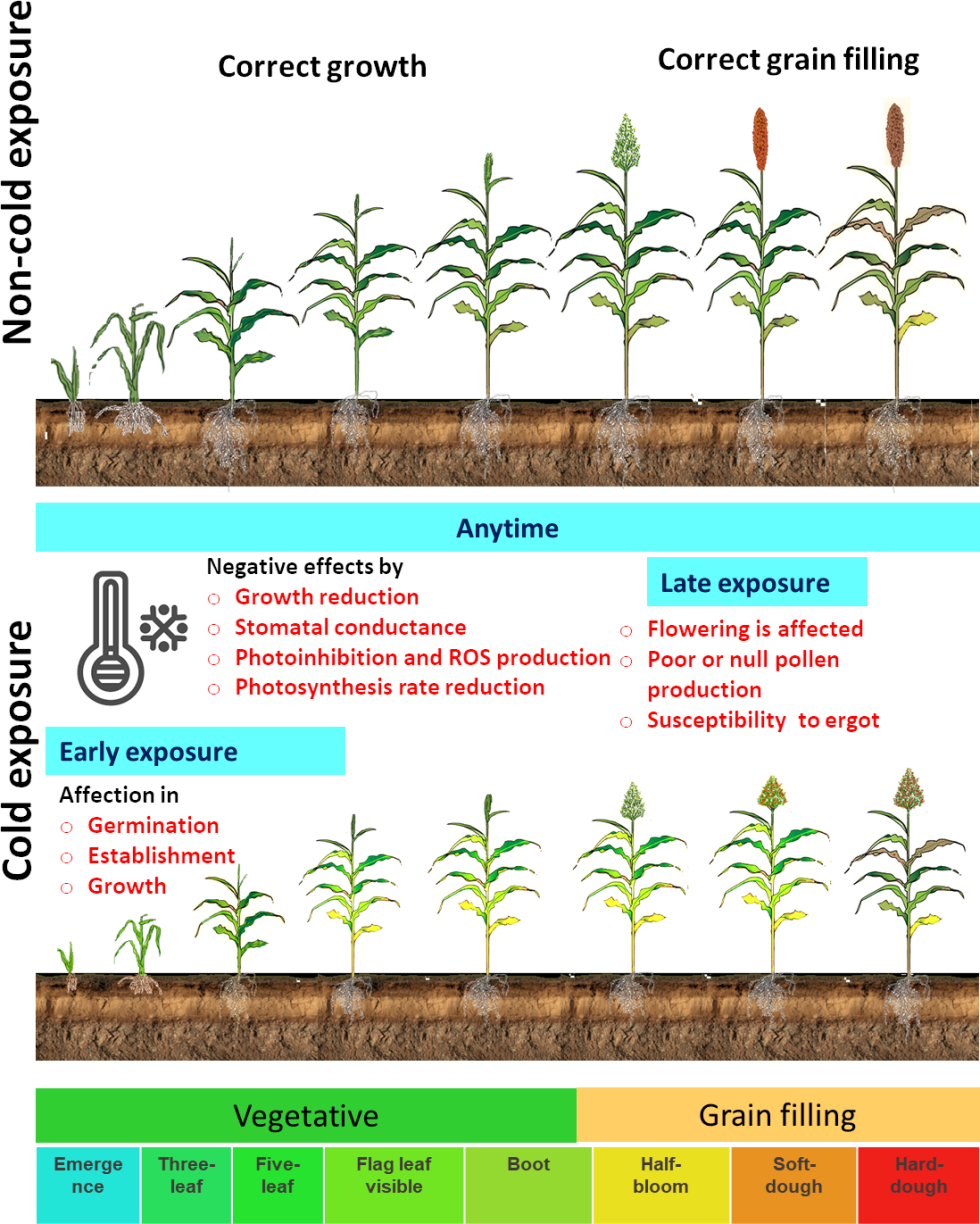
Diagrammatic illustration of sorghum development and the cold exposure effect in sorghum. The principal growth stages are used to represent the effect in early, late, or any time of exposure to cold (Ostmeyer et al., 2022).

The molecular mechanisms that respond to cold temperatures are different from freezing (<0°C) in comparison with chilling stress (0°C to 15°C). During freezing, the physical state changes in membrane lipids, leading to cell or tissue injury, and this explain why cold-tolerant sorghum varieties are still affected by freezing temperatures (Palta and Weiss, 2018). Low temperatures decrease germination, emergence rate, seedling establishment, plant growth and development, root development, and biomass accumulation (Ercoli et al., 2004; Chinnusamy et al., 2007; Janmohammadi et al., 2015; Ortiz et al., 2017; Hu et al., 2022). The cold stress reaction in sorghum varies depending on the temperature level, duration, and phenological stage. In sorghum development, cold exposure could be in the early growth stages or in the late growth stages (**Figure 1**). The susceptible sorghum cultivars exposed to cold temperatures in early growth stages (i.e., when sorghum is sown at the beginning of the spring) show poor or no germination. Cold stress negatively affects germination, establishment, and growth, reducing sorghum biomass production and grain yield (**Figure 1**) (Knoll and Ejeta, 2008; Knoll et al., 2008; Maulana et al., 2017; Parra-Londono et al., 2018). Under continuous or late exposure to cold stress, susceptible genotypes can, sometimes, grow to vegetative phenological stages, but reproductive phases are affected. In some cases, flowering does not occur, and pollen production is reduced or even abolished (Osuna-Ortega et al., 2003). The young microspore stage is especially susceptible to cold, and male sterility is affected by cold, causing a massive grain yield loss. In addition, late or absent pollen production during flowering under cold conditions favors ergot (*Claviceps africana*) infection in cold-sensitive varieties (Hernández-Martinez et al., 2008).

Low temperatures can lead to tissue dehydration and water deficit by reducing water uptake without reducing the leaf transpiration rate (Aroca et al., 2012; Bekele et al., 2014). The development of the root system can be influenced by low temperatures, which can affect water and mineral acquisition (Huang et al., 2005). Studies have shown that root elongation in sorghum seedlings is mostly determined by temperature and not by the diurnal cycle (Iijima et al., 1998). Some studies emphasize the role of root development in chilling survival in plants (Melkonian et al., 2004; Matsumoto et al., 2009). Bekele et al. (2014) found that root establishment was the most important factor, affecting plant survival under chilling stress. Under low-temperature conditions, extensive phenotyping of the root architecture of sorghum seedling showed that the root-to-shoot ratio, root biomass, and root length were correlated with the chlorophyll content (Bekele et al., 2014). Furthermore, it was found that root length and biomass developed at optimum temperature were correlated with chilling survival, reflecting the influence of root biomass on cold survival rather than only on primary root length (Bekele et al., 2014). Thus, root establishment was the most critical factor, affecting field establishment and survival under prolonged chilling stress conditions.

Sorghum–rhizosphere interactions can be a crucial component to consider when breeding this cultivar for cold stress tolerance. Numerous studies have focused on the effects of root exudates in determining the integration of the rhizosphere (Schlemper et al., 2017; Wang et al., 2021) and how these root microbiomes can mitigate different sorts of abiotic stresses (Wu et al., 2021; Yukun et al., 2021). Plant growth microorganisms that have potential to promote plant growth at low temperatures are a worldwide trend in the field of agricultural inoculation technology (Malusá et al., 2012; Pandey and Yarzábal, 2018). Cloutier et al. (2021) studied how the sorghum genotypic affected the root rhizosphere community and how these associations were affected by frost stress. They found that plant genotype influences root flavonoids and the rhizosphere community composition and that these relationships are affected by frost (Cloutier et al. 2021). The analysis of root hydraulic conductance and transpiration rate differences, as well as the analysis of varieties with different tolerance to cold stress, should provide a novel insight into the root contribution to chilling tolerance in sorghum and the genetic mechanisms implicated (Bekele et al., 2014).

## Physiological and biochemical response to cold stress in sorghum

### Reduction or cessation of growth in sorghum

Critical cellular functions such as photosynthesis, respiration, and membrane permeability are affected by cold temperatures (Rawat et al., 2021). Cold acclimation involves many physiological and biochemical changes, the principal changes being the reduction or cessation of growth (Achard et al., 2008). The reduced sorghum growth can be explained by the susceptibility of its C4 photosynthetic machinery to low temperatures, which, at the same time, can be modulated by the upregulation of C-repeat binding factor/dehydration-responsive element binding (*CBF/DREB*) that are required to activate cold-responsive genes, causing growth inhibition. These mechanisms have been studied in other plants, including Arabidopsis and rice (Ito et al., 2006). The mechanism of growth inhibition also involves the stimulation of gibberellin-inactivating enzymes in a process that promotes survival or escape from adverse environmental conditions (Achard et al., 2008). The reduction in growth could also be caused by an increase in reactive oxygen species (ROS) in plants, which affects growth and reduces crop yield (Devireddy et al., 2021; Wang et al., 2022).

### Stomata aperture and closing

Cold-sensitive plants usually have low leaf water potentials, whereas cold-tolerant plants preserve water potentials by closing their stomata and preventing water loss through transpiration during chilling (Wilkinson et al., 2001). Stomatal conductance and transpiration rates have been suggested as traits that can be used for marker-assisted selection in sorghum (Ortiz et al., 2017). The ability of the plants to maintain gas exchange is vital for biomass production (Khanthavong et al., 2022). Under waterlogging and low temperature, the environmental responses of stomatal conductance in sorghum showed a decrease in stomatal conductance, compared with the combination of moderated soil moisture or gradual soil drying with high temperature (Khanthavong et al., 2022).

### Photosynthesis reduction to chilling temperatures

Light and water use are more efficient in C4 plants than that in C3 plants (Zhu et al., 2008b). However, only a few C4 plants are able to maintain photosynthetically competent leaves at chilling temperatures (<15°C) (Du et al., 1999; Wang et al., 2008). The ability for active photosynthesis during exposure to cold is vital for sorghum or any other crop bred for cold tolerance (Hurry et al., 2002). The leaf greenness indicates photosynthetic activity, which is a necessary trait for selection of chilling tolerance.

Under cold conditions, pyruvate phosphate dikinase (PPDK) instability is associated with a reduced photosynthetic rate in leaves (Naidu et al., 2003; Wang and Li, 2010). PPDK is a cold-labile enzyme that plays a critical role in the carboxylation of pyruvate to phosphoenolpyruvate in C4 metabolism plants. However, some C4 plants such as *Miscanthus giganteus* are adapted to cold conditions (Naidu et al., 2003), which has a high level of expression of ribulose-1,5-bisphosphate carboxylase/oxygenase (Rubisco) and PPDK under cold stress, contrasting to maize and other cold susceptible C4 plants (Ortiz et al., 2017). The reduction in Rubisco activity under low temperatures is another limitation to carbon assimilation in C4 plants compared with the photosynthetic rates of C3 plants (Sage, 2002). The lower quantum yield of photosynthesis (ΦPSII) under cold stress indicates a reduction in the linear electron transport rate (Ortiz et al., 2017). Genetic variation in the photosynthetic response, the ability to recover, and the carbon fixation capacities of sorghum exposed to cold stress has been reported (Ortiz et al., 2017). Further analysis of sorghum varieties could include the selection of varieties with better carbon fixation under cold conditions, helping to improve yield and biomass.

## Photoinhibition and production of reactive oxygen species

The chloroplasts and peroxisomes are the primary sources of ROS generation in light exposition on the mitochondria in the darkness (Foyer and Noctor, 2003; Singh, 2010). The low efficiency of PSII affects excitation energy transfer, leading to photoinhibition and ROS production (Rochaix, 2014). Chilling stress and light can lead to photoinhibition, a phenomenon in which energy transfer from chlorophyll to oxygen is unbalanced. This can produce toxic ROS (Pimentel et al., 2005). During abiotic stress, ROS have a double role; they are toxic at high levels, but, at appropriate levels, it can activate plant defense systems, which are harmful to cells under cold stress (Nadarajah, 2020). ROS can cause membrane damage by lipid peroxidation, proteins, and DNA (Ahmad et al., 2008; Das and Roychoudhury, 2014). In intracellular signaling, ROS act as second messengers to elicit tolerance of abiotic and biotic stresses (Ben Rejeb et al., 2014).

Sorghum has developed mechanisms to prevent the production of ROS and, once formed, to detoxify and repair the damage (Mansour et al., 2021). Sorghum can reduce light absorption in two ways: photoreceptors can sense bright light and move the chloroplast away from the light (Jarillo et al., 2001; Kodama et al., 2008); and also some carotenoid, such as zeaxanthin and antheraxanthin, can dissipate the excess of absorbed light energy (Sharma and Hall, 1996) and inhibit lipid peroxidation (Niyogi et al., 1997). In addition, sorghum has antioxidant machinery that can scavenge ROS composed of enzymatic constituents such as superoxide dismutases (SODs), catalases, and glutathione reductases, as well as antioxidant compounds such as ascorbic acid and flavonoids (Dykes and Rooney, 2006). It has been reported that cold stress increases the activity of SODs and catalases (Petrić et al., 2013).

## Metabolic changes in response to cold in sorghum

**Figure 2:**
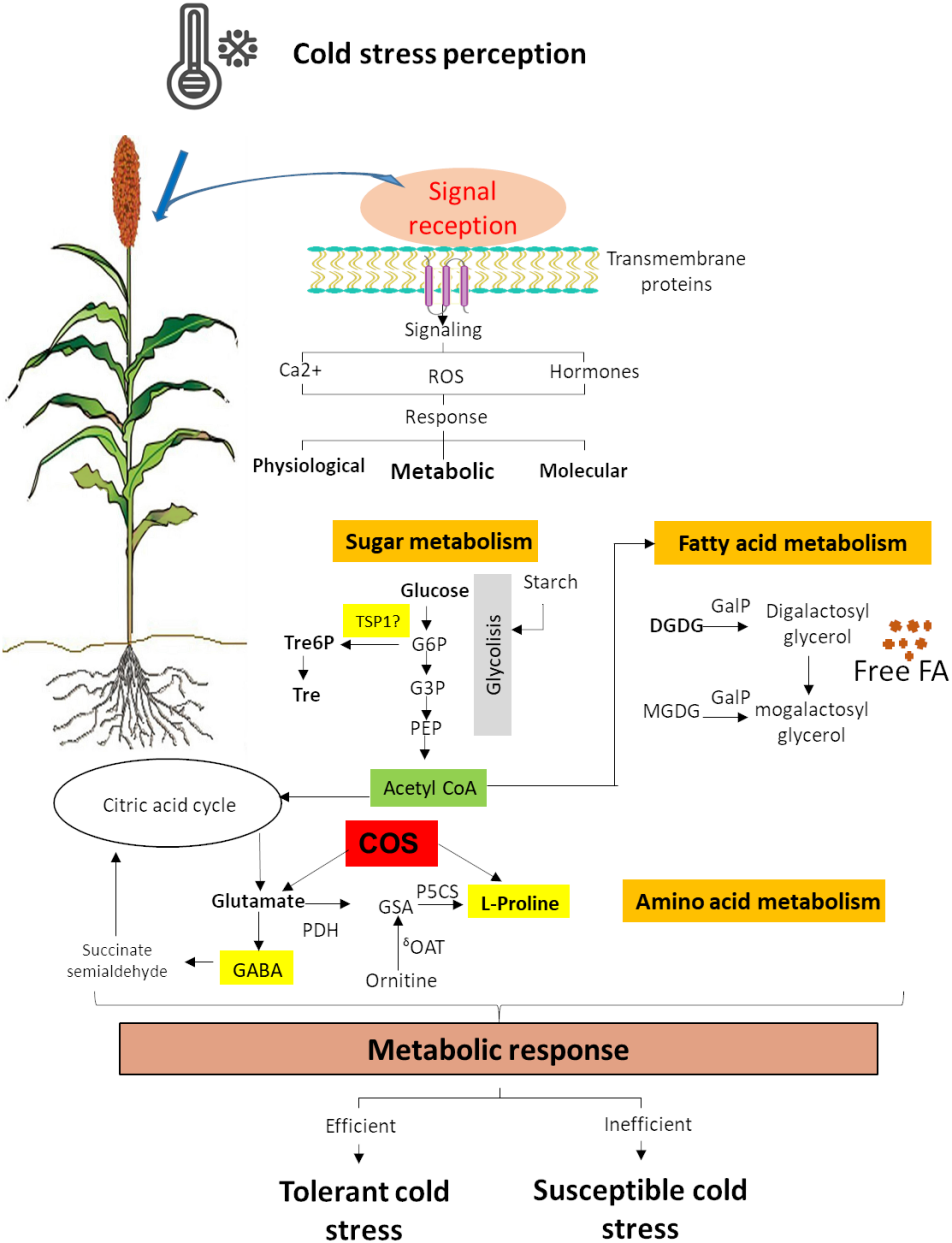
Sugar, amino acid, and fatty acid metabolism response to cold stress. G6P, D-glucose-6-phosphate; G3P, D-glucose-3-phosphate; Tre6P, trehalose-6-phosphate; Tre, trehalose; TSP1, alpha-trehalose-phosphate synthase; COS, chitooligosaccharides; GSA, glutamatesemialdehyde; P5C, L-1-pyrroline-5-carboxylate; GABA, γ-aminobutyric acid; P5CR, P5C reductase; P5CS, glutamate 5-kinase; GDH, glutamate dehydrogenase; ^δ^OAT, ornithine aminotransferase; MGDG, monogalactosyldiacylglycerol; DGDG, digalactosyldiacylglycerol; GAlP, galactolipase; FA, fatty acid (Ostmeyer et al., 2022).

Cold acclimation comprises many biochemical changes, including the accumulation of cryoprotective molecules (Achard et al., 2008; Rani et al., 2021) such as several sugars, lipids, and nitrogenous compounds. These changes are the result of metabolic response (**Figure 2**) (Li et al., 2018; Vera-Hernández et al., 2018; Lin et al., 2019, Yu et al., 2022), which provides an excellent tool to improve the stress tolerance of this important crop.

### Sugars in cold stress

Cold stress enhance the accumulation of soluble sugars, which are originated from starch metabolism (**Figure 2**) (Lin et al., 2019; Shi et al., 2022). Sugars are an energy source, but they also have other functions as substrates for polymers, carbon precursors, storage as reserves, and signaling molecules during cold stress (Yamada and Osakabe, 2018). Trehalose and trehalose-6-phosphate are disaccharides that have a protective role in proteins and membranes during abiotic stress (Elbein et al., 2003; Iordachescu and Imai, 2008). Sorghum transgenic lines with overexpression of alpha-trehalose-phosphate synthase (*TSP1*) were able to tolerate high salinity and develop higher root growth and biomass (Yellisetty et al., 2015). Still, tolerance to cold stress has not been tested yet. Rice seedlings treated with chitooligosaccharides (COS) showed cold tolerance and increased contents of proline and glutamate, revealing that COS significantly induced genes associated with the glutamate and proline biosynthesis pathway (Zhang et al., 2019) (**Figure 2**). This suggests that COS might be a key regulator through the accumulations of glutamate and proline (Zhang et al., 2019) and that it could be explored in sorghum. Glucose and fructose levels increased under chilling in sorghum, and sucrose was slightly lower (Marla et al., 2017). The small starch content and the increase in simple sugars during chilling exposure to sorghum suggest an essential role for the B-amylases in cold acclimation (Marla et al., 2017).

### Lipids in cold stress

Membrane lipids remodeling is key for cold tolerance. The fatty acid unsaturation helps preserve membrane integrity during chilling by lowering the melting temperature and increasing fluidity (Marla et al., 2017). The ability to adjust membrane fluidity to low temperature change is associated with regulating membrane fatty acid desaturation. The desaturation of both phospholipids and sphingolipids is essential for remodeling the plasma membrane under cold stress (Barrero-Sicilia et al., 2017). In chilling-sensitive plants at low temperatures, the galactolipase (GAlP) is significantly active, leading to an increase in free fatty acid in chloroplasts (**Figure 2**) (Kaniuga, 2008).

Chilling causes a decrease in the concentrations of mono-galactosyl-diacyl-glycerols (MGDGs) and sulpho-quinovosyl-diacyl-glycerols (**Figure 2**), which are significant lipids in the thylakoid (Marla et al., 2017; Liu et al., 2018). Lipid metabolism gene analysis in sorghum has identified lipids deregulated in response to chilling in sorghum seedlings that may cause chilling tolerance in this crop (Marla et al., 2017). Genes that are involved in fatty acid biosynthesis (i.e., acetyl coenzyme A (CoA) synthetase and acyl-Acetyl coenzyme A (CoA) oxidase 1), thylakoid lipid synthesis (MGDG synthase MGD2 and DGFGI synthase 1 DGD1), and phospholipids metabolism (G-3-phosphatase acyltransferase 5, phosphatidiylserine synthase, and phospholipase D delta) were upregulated in response to chilling (Marla et al., 2017). An increase in the unsaturation index of total phospholipid PC and LysoPC also has observed under chilling in sorghum (Marla et al., 2017).

### Nitrogenous compounds in response to cold

During cold stress, certain amino acids and amine compounds adjust its metabolism, particularly those associated with proline biosynthesis (**Figure 2**) (Kaplan et al., 2004). The proline amino acid can act as a regulatory or signaling molecule capable of altering the expression of antioxidants-associated genes in plant response to environmental constraints (De Carvalho et al., 2013; Ben Rejeb et al., 2014). During dehydratation, proline accumulates and contributes to ROS formation in mitochondria, playing a role in the hypersensitive response in plants (Ben Rejeb et al., 2014). Proline is usually used as an osmolyte, but it can also function as a powerful antioxidant (Das and Roychoudhury, 2014). Proline accumulation is an adaptive mechanism that allow plants tolerate cold or chilling stress (Teh et al., 2016). Proline accumulation in stress situations will depend on both the activation of its biosynthesis and the inhibition of its degradation (**Figure 2**) (Ben Rejeb et al., 2014). In cold-tolerant sorghum plants, a higher proline accumulation is related to a better tolerance (Vera-Hernández et al., 2018). There are two biosynthesis pathways for proline, glutamate, and ornithine (**Figure 2**). The enzymes Δ^1^-pyrroline-5-carboxylate synthetase (P5CS), Δ^1^-pyrroline-5-carboxylate reductase (P5CR), and ornithine-δ-aminotransferase (δ-OAT) are involved in the proline biosynthesis (**Figure 2**) (Bagdi et al., 2015; Vera-Hernández et al., 2018). A transcription factor binding site analysis of these genes indicated the presence of a low-temperature response element in the promoter region of ɗ-OAT, suggesting that the proline accumulation in cold response in sorghum may be using the Orn pathway (Vera-Hernández et al., 2018). *P5CS2* expression is associated with high proline levels in response to drought stress in sorghum, resulting in an exciting gene for cold tolerance analysis. γ-Aminobutyric acid (GABA) is another amine metabolite that can be rapidly accumulated in plant tissues and is associated with cryoprotection in monocots (Mazzucotelli et al., 2006), which could be involved in tolerance to chilling stress in sorghum (**Figure 2**).

## Molecular response to cold stress in sorghum

### Transcription factors and proteins involved in cold stress response

**Figure 3:**
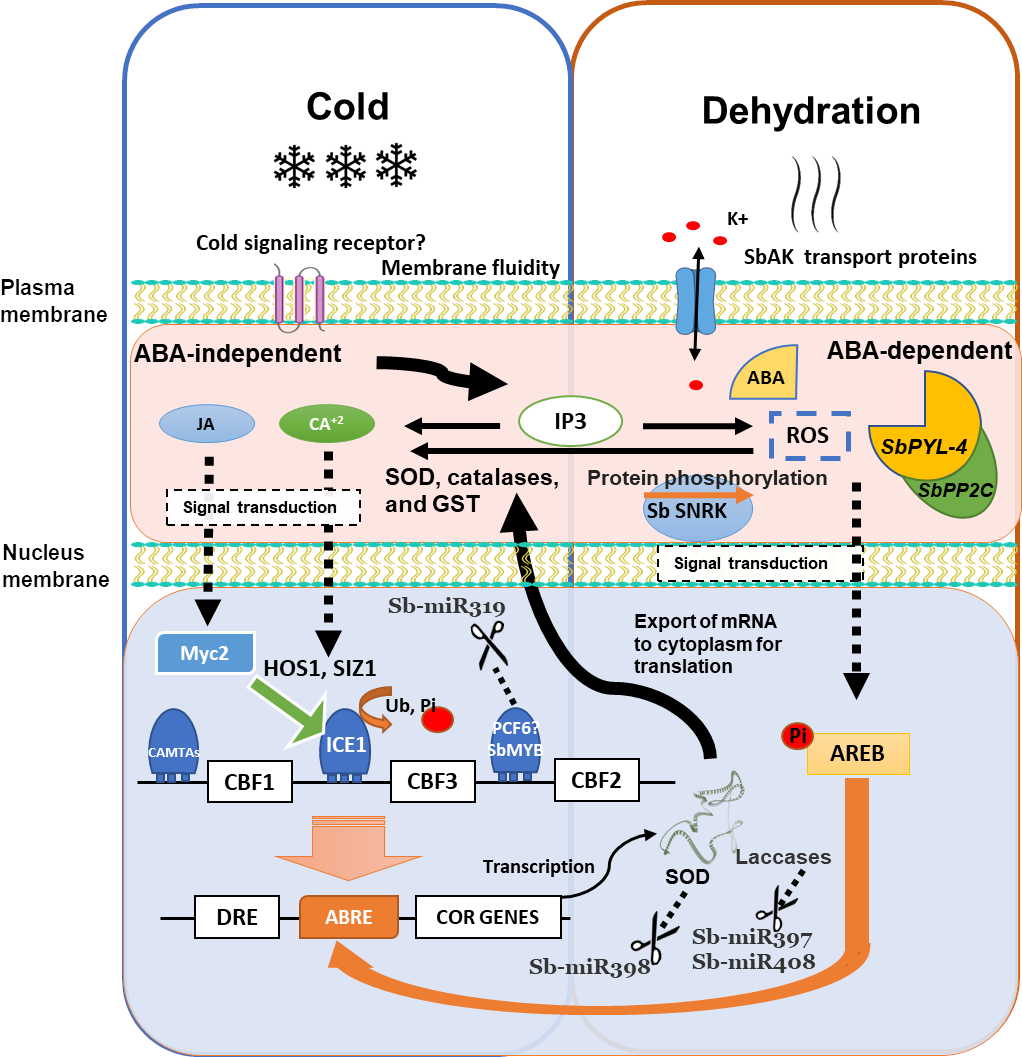
Schematic overview of the cold response and its overlapping dehydration response–mediated regulatory networks in Sorghum. Cold may be perceived by signaling receptor and the second messengers (IP3, Ca2+, and ROS), transducing signals through protein kinases or TF cascades. *CBF*s are activated by *ICE1* and *CAMTA* TF. *HOS1* and *SIZ1* regulate ICE1 protein. Cold stress activates *ICE1*, and *AREB* is induced by ABA. The DRE element is recognized by *CBF1* and control *COR* genes. MiR398, miR397, and miR408 regulate some *COR* genes. The JA signaling pathway activates *MYC2*, which can activate the expression of *ICE* in *ICE-CBF-COR* cold resistance pathway. ROS, reactive oxygen species; IP3, inositol 1,4,5-triphosphate; CPK, calcium-dependent protein kinase; MAPK Ras, mitogen-activated protein kinase; Pi, phosphoryl group; miR, microRNA; SOD, superoxide dismutase; GST, glutathione S-transferase; *CBF1*, C-repeat binding factors; Ub, ubiquitin; SUMO, small ubiquitin–related modifier. Modified by Shi and Yang (2014).

Transcription factors (TFs) are a category of proteins that bind to cis-regulatory DNA sequences and are responsible for either positively or negatively affecting the transcription of certain genes. They determine whether a specific gene will be turned “on” or “off” (Philips and Hoopes, 2008). Currently, the plant’s most understood cold-signaling pathway is the *CBF/DREB* transcriptional regulatory cascade (Shi et al., 2015; Wang et al., 2022). The *CBF* genes respond rapidly to chilling temperatures and induce the expression of several proteins that protect plants from freezing temperatures. These proteins play a crucial role in cold acclimation (Agarwal et al., 2006; Nakashima and Yamaguchi-Shinozaki, 2006; Achard et al., 2008). Sb*CBF6* is highly expressed during chilling in tolerant sorghums (Chopra et al., 2015; Marla et al., 2017). *CBF* expression is controlled through jasmonic acid (JA) (**Figure 3**). In this sense, allene oxidase synthase and 12-oxoyphytodienic acid reductase, which are important genes in JA biosynthesis, are upregulated in chilling-tolerant sorghum. This suggests that JA biosynthesis contributes to chilling tolerance (Marla et al., 2017). DELLA proteins (a critical negative regulator of gibberellin signaling) may contribute to the CBF1-mediated freezing tolerance (Achard et al., 2008). *DREBs* (a significant subgroup of *AP2/ERF* TF) induce the expression of genes involved in abiotic stresses. Genes that code for DREB proteins regulate the transcription of many genes involved in the plant response to cold stress. Fifty-two *ERF* (ethylene response factor) genes encoding DREB proteins have been predicted in sorghum (Mathur et al., 2020). Bioinformatic analysis showed that the motifs within sorghum DREB promoters are principally involved in abscisic acid (ABA), light, and calcium-mediated regulation (Srivastav et al., 2010). However, the regulation of *DREB* genes is not well understood in sorghum. In sorghum, the CBF/DREB and ERF proteins have similar structures and evolutionary relationships (Mizoi et al., 2012; Rashid et al., 2012). In sorghum, CBF/DREB and ERF proteins have groups that contain an LWSY motif at the C-terminus, which respond to cold treatment, suggesting that such motif may be involved in cold plant tolerance (Yan et al., 2013). Sorghum genes that are predicted to be involved in ethylene biosynthesis, which regulates cold signaling pathways, were upregulated (Marla et al., 2017). Recently, it was indicated that *TaMYC2* plays a critical role in cold stress in many plants and activates the *ICE* (inducer of CBF expression)*–CBF-COR* (cold responsive) cold resistance pathway through interaction with *ICE* in Arabidopsis (Wang et al., 2022), and some *MYC2* (A/B/D) improved cold tolerance through ROS metabolic.

Another critical group of TF is *NF-Ys* (plant nuclear factors), which show differential stress response gene expression in several tissues and interact with abiotic stress signaling pathways to regulate the plant stress responses (Kavi et al., 2022). In sorghum, 24 *NF-Y* genes are expressed in cold stress (Maheshwari et al., 2019). Sixteen *NF-Ys* (*NF-YA*2/4/6/8, *NF-YB2*/7/10/11/12/14/16/17, and *NF-YC*4/6/12/13) are induced by both cold and high temperatures (Maheshwari et al., 2019). These suggest a crosstalk among both stresses and microRNA (miRNA) association in the regulation of Sb*NF-Ys* (Maheshwari et al., 2019), denoting that miRNAs may also participate in gene networks controlled by TFs like *NF-Y*s (Maheshwari et al., 2019).

ABA is an important hormone implicated in the cold stress response by regulating specific stress-responsive genes (Xue-Xuan et al., 2010). The genes that are predicted to be involved in the biosynthesis of ABA were upregulated in sorghum during cold treatment (Marla et al., 2017). Regarding the amino acid sequences of *ABA* receptor (*ABAR*s) family genes from Arabidopsis, eight candidate genes were identified in the sorghum genome (Dalal and Inupakutika, 2014). ABA binds to *ABAR*s and brings conformational changes that facilitate ABA-mediated interaction of PYL with PP2C. Dalal and Inupakutika (2014) identified these eight genes as members of the PYL family in sorghum, namely, *SbPYL1–SbPYL8*. In addition, nine functional *SbPP2C* genes were predicted in the sorghum genome (Dalal and Inupakutika, 2014). Among *ABAR*s receptors, the *SbPYL4* gene was found to be specific for cold stress, with higher expression observed in leaf exposed to cold stress. *PhyA* and *phyB* genes have been reported to function antagonistically to regulate cold tolerance through ABA-dependent jasmonate signaling (Yang et al., 2019). In tomatoes, the genes HY3 and HY5 enhance cold tolerance by integrating myoinositol and light signaling (Yang et al., 2019). Lin et al. (2021) found that HY5 can be activated by photoreceptors to promote photomorphogenesis (Lin et al., 2021).

During chilling, genes encoding components of photosystem I (PSI/P700) and photosystem II (P680), responsible for the light reactions phase of photosynthesis, are downregulated (Marla et al., 2017). Sobic.003G370000, an ortholog of At*NPQ4* (nonphotochemical quenching), was increased under chilling and could be implicated in preventing damage to the photosynthetic apparatus due to photoinhibition (Marla et al., 2017), as was reported in Arabidopsis (Havaux et al., 2000). ROS detoxification in plants has been mediated by the ascorbate-glutathione cycle (Foyer and Noctor, 2011). Marla et al. (2017) showed that the monodehydroascorbate reductase 1 (Sobic.007G171000) and the ascorbate biosynthesis gene vitamin C defective 5 (VTC5, Sobic.008G064700) of the ascorbate–glutathione cycle were highly expressed during cold stress in sorghum. Similarly, some glutathione S-transferase (GST) enzymes have antioxidant properties. Some GST genes were highly upregulated in chilling-tolerant sorghum but not in chilling-sensitive sorghum in cold treatments. Other genes involved in sugar breakdown as hexokinase (Sobic.009G06980) and fructokinase (Sobic.003G386000) increased during cold in sorghum. Several starch synthase genes were induced, which correlated with the composition of the carbohydrate in sorghum during cold stress (Marla et al., 2017).

C2H2-type zinc finger proteins are one of the best-studied TF associated with abiotic stress in plants (Liu et al., 2022). In *S. bicolor*, 145 Sb*C2H2-ZFP* members have been predicted (Cui et al., 2022). In sorghum, Sobic.005G121100 was significantly upregulated in cold stress, and Sobic.008G088842 was activated by cold and inhibited in drought in stems and leaves (Cui et al., 2022). In tomatoes, multiple B-box proteins (BBxs) play a role in responses to light quality and cold stress (Bu et al., 2021). In SlBBX7-, SlBBX9-, and SlBBX20-silenced tomato plants, cold tolerance was suppressed. Moreover, Bu et al. (2021) found a photosynthetic response instantly after cold stress by the impairment of non-photochemical quenching, and the consequent excess photon energy excited by low temperature is not consumed, leading to the over-reduction of electron carriers and damage of the photosystem (Bu et al., 2021). Twenty-four Sb*BBX* genes have been identified in sorghum (Shalmani et al., 2019), suggesting that Sb*BBX* studies in response to cold tolerance may improve the current understanding of this trait.

Differential expression of K+ transport genes might also be involved in sorghum’s cold response. Anil Kumar et al. (2022) found higher expression levels of potassium transport genes (Sb*AKT1*, Sb*HAK7*, Sb*HKT5*, Sb*HAK25*, and Sb*AKT7*) in cold stress treatments. In this context, genes with differential expression could be proposed as good candidates for functional analysis and further use in genetic engineering, and traditional breeding programs increase the cold tolerance of sorghum.

Heat shock proteins (HSP) function as chaperons and protect proteins from the harmful effect of different types of abiotic stress besides heat, including cold (Sabehat et al., 1998; Swindell et al., 2007). Transcriptomic analysis showed that the HSP Sb03g027330 was highly abundant under cold stress (Chopra et al., 2015). GST enzymes have functions in detoxifying xenobiotic compounds and ROS and are abundant under cold stress in sorghum (Chopra et al., 2015). Late embryogenesis abundant proteins are essential for membrane stabilization when the cytoplasm becomes dehydrated (Hanin et al., 2011) and might be expressed under cold conditions by a crosstalk signaling process with ABA (Li et al., 2017). To understand better how cold stress signals are perceived and integrated into gene regulation by changes at multiple levels, it is necessary to know the TFs, miRNAs, and the expression of gene products that might be involved in different degrees of tolerance to low temperatures, resulting in a change in metabolites in plants.

### miRNAs involved in the repression of genes in cold stress

miRNAs are small non-coding RNAs, approximately 18–24 nucleotides in length, that can control gene expression at the post-transcriptional level (Fire et al., 1998). miRNAs act as regulators of gene expression through the degradation or inhibition of target gene translation (Djami-Tchatchou et al., 2017). The upregulation of miRNAs is associated with reduced expression of its target gene (Megha et al., 2018). In contrast, the downregulation of a miRNA can increase its target genes expression. MiRNAs may mediate the gene regulation network under cold stress in two ways: regulating stress-related signal transduction pathways or modulating the expression of cold-responsive TFs (Huang et al., 2022). Target genes and miRNAs involved in cold stress responses as a complex gene network may influence cold tolerance.

Some miRNAs participate in plant responses to cold stress (Thiebaut et al., 2012; Liu et al., 2018; Martínez Núñez et al., 2021; Satyakam et al., 2022). MiR397 is involved in different abiotic stress, including cold (Huang et al., 2020). For example, under normal conditions, miR397 is expressed at high levels, altering the abundance of its target genes laccases, which play an essential role in anthocyanin biosynthesis and abiotic stress responses (Xu et al., 2022; Zaman et al., 2022). Under cold stress conditions, miR397 is downregulated, leading to the accumulation of laccases (Xu et al., 2022). This association between miR397 and its target genes under cold treatments has been observed in plants including wheat (Gupta et al., 2014) and grapevine (Sun et al., 2015). In addition, miR397 overexpression improved the tolerance to cold stress in Arabidopsis (Dong and Pei, 2014; Satyakam et al., 2022). The core regulator in cold acclimation *ICE1* (Chinnusamy et al., 2007) was identified as a target of miR397 in cold adaption in wheat. In sorghum, a homolog of miR397 was identified (Dash et al., 2022).

MiR319 and its target genes have been analyzed in the monocot sugarcane under cold stress (Thiebaut et al., 2012). Thiebaut et al. (2012) found differences in the timing and intensity of regulation of miR319 and its target genes *PCF5*, *PCF6*, and *GAMyb*. The Myb family genes are known to be *CBF*s suppressors; therefore, the upregulation of miR319 can prevent its repression effect, leading to the expression of *COR* genes (**Figure 3**), possibly contributing to cold tolerance. The overexpression of miR319 can enhance cold tolerance, after chilling acclimation, in transgenic rice seedlings (Yang et al., 2013; Wang et al., 2014; Iqbal et al., 2021). Another example of this is the family of miR398; they control Cu/Zn SODs under oxidative stress caused accumulation of ROS during cold or some other kind of abiotic stress such as heat and salinity stress. SODs play an essential role in converting superoxide to H_2_O_2_ and molecular oxygen and thus reduce oxidative stress in plant cells. Downregulation of miR398 is vital for the SODs expression and ROS detoxifying. This association between miR398 and its target genes has been observed in cold-stress plants, including Arabidopsis (Sunkar and Zhu, 2004) and grapevine (Sun et al., 2015).

In sorghum, miRNA–target gene interactions generated with degradome analysis have been reported in cold response, and some interactions as miR169-*NF-Y* network were identified (Franke et al., 2018). Validation of miRNA–target gene networks will screen their roles in cold stress tolerance, which will also help develop plants with stress tolerance.

### Networks involved in cold stress response in sorghum

Understanding the mechanisms of cold stress response at the molecular level is crucial for improving crops against stresses without affecting the yield (Adhikari et al., 2022). Integration of signal perception, signal transduction, gene expression, and molecules produced is essential to understand the cold stress response and cold plant acclimation. Several molecules, including calcium (Ca2+), receptors, ROS, and signaling proteins, have been identified in plants. TFs, miRNAs, and proteins also play an essential role, and several have been identified in sorghum’s cold response. In summary, an illustration of the cold response and its overlapping dehydration response–mediated regulatory networks in sorghum is proposed using the evidence previously described (**Figure 3**).

The mechanism of sensing low temperatures in plants is scarce, and it is still unknown whether there is a cold signaling receptor; possibly, with a similar complex as in rice (Ma et al., 2015), Chilling Tolerance Divergence1 (*COLD1*) encodes a regulator of G protein signaling coupled with rice G protein A subunit 1 that participates in cold stress signaling *via* Ca2+ signals (Ma et al., 2015; Zhu et al., 2022), regulating the cold stress–driven influx of intracellular Ca2+. Low temperatures can change the membrane fluidity; the potential sensors of cold include Ca2+ influx channels, transmembrane stress-sensing histidine kinases proteins (Fedurayev et al., 2018), and receptors associated with G proteins (Zhu et al., 2022), which could allow identify the low temperatures and induce a signal to subsequently transduce it to the nucleus (Chinnusamy et al., 2007). A decrease in cell membrane fluidity leads to conformational changes in membrane proteins and lipids, which generate second messengers IP3, Ca2+, and ROS, which are necessary to transduce signals through protein kinases or TF cascades (**Figure 3**). Ca2+ is the most common secondary messenger in plants. Within only a few seconds under low temperatures, cytosolic Ca2+ can liberate from vacuoles. The Ca2+ release is upstream to the expression of *CBFs* and *COR* genes in the cold signaling pathways (Hiraki et al., 2019); in the same manner, ROS and IP3 serve as second messengers to be integrated into a genetic response that initiates variations in gene expression, leading to physiological and metabolic changes in the cell, and culminates in response and tolerance (Pareek et al., 2017).

Plant hormone JA also affects the low-temperature stress response. The JA signaling pathway activates *MYC2*, which is involved in most JA-mediated responses; in the same way, *MYC2* is capable of activating the expression of *ICE in ICE-CBF-COR* cold resistance pathway. *CBF*s and *AREB* TFs regulate *COR* genes containing *CRT/DRE* and *ABRE* motifs in their promoters, respectively (**Figure 3**). *CBF*s are activated by *ICE1* and calmodulin-binding transcription activators (*CAMTA)* TFs but are suppressed by *Myb* family TFs. MiR319 can suppress *TCP*-like and *MYB*-like factors, stopping its repression effect and possibly contributing to cold tolerance. *HOS*1 and *SIZ1* encode protein ligases that regulate the abundance of ICE1 protein (Zhou et al., 2011). Cold activates ICE1, and AREB is induced by ABA-mediated dehydration signaling. In response to cold stress, *DRE* element is recognized by *CBF1 TFs*, which control the expression of COR genes. At the same time, some COR genes are regulated by miRNAs, such as SODs by miR398, and laccases by miR397 and miR408. Consequently, the downregulation of these miRNAs is necessary for COR genes expression. There is a correct coordination between the different elements, and different elements are conserved in both biotic and abiotic stress responses. The more information that we have, the more precise the mechanisms of response and tolerance in sorghum will be.

### Genomic mapping, chromatin remodeling, and epigenetic memory in sorghum

The sorghum genome of ~730 Mb has been sequenced (Paterson et al., 2009). Quantitative trait locus (QTL) is the most comprehensive tool for marker‐assisted selection. In sorghum, co‐localization of different QTLs close to each other is known as hotspots, highly heritable genes map irregularly to the 10 sorghum chromosomes, and several are physically clustered together in chromosomes. In parallel, there are other vital tools, such as cold stress–induced transcriptome profiling (Chopra et al., 2015) and analysis of single-nucleotide polymorphisms (Chopra et al., 2015; Parra-Londono et al., 2018). Cold acclimatization is a process conserved between species where many genes are induced (Chinnusamy et al., 2007). Histone marks like acetylation and deacetylation are central for activation and repression during cold acclimation. The *HOS15* gene product, which works as a histone deacetylation, interacts explicitly with histone H4 during cold acclimatization in Arabidopsis (Zhu et al., 2008a; Park et al., 2018). The chromatin remodeling derived from epigenetic changes during cold acclimatization has not been studied yet in sorghum. Nevertheless, there are some clues about epigenetic memory to cold stress; sorghum exposed to photoinhibition treatment changes the levels of the carotenoids and then restores during a recovery period, and, when photoinhibited, plants showed better protection (Sharma and Hall, 1996). 

## Sources and screening for cold-tolerant genotypes of sorghum

Some genetic sources of cold tolerance in sorghum have been identified (Singh, 2010; Burow et al., 2011; Woldesemayat et al., 2018). Genetic variability between sorghum genotypes for traits associated with early-stage chilling tolerance plays an essential role in future research (Rutayisire et al., 2021). One of the most studied sources of cold tolerances is Chinese landraces, showing a higher emergence and seedling vigor compared with commercial lines, under controlled and field cold conditions. Some undesirable characteristics are associated with cold-tolerant sorghums, particularly grain tannins and tall plants (Franks et al., 2006).

Cold tolerance in sorghum has been assessed by different traits such as germination (Tiryaki and Andrews, 2001) and seedling establishment and vigor (Upadhyaya et al., 2015) under low temperatures. The use of methods based on a rank summation index of traits such as emergence index, percentage, shoot and root dry weight, seedling height, and vigor score during growth under low temperature has been described (Yu et al., 2004; Rutayisire et al., 2021). Most of the research has focused on developing early-season cold-tolerant genotypes (Emendack et al., 2021). By planting early mature varieties and manipulating the sowing date, severe damage can be avoided in the early cold exposure conditions; however, the development of cold-tolerant varieties is necessary to deal with late or prolonged exposure to cold. Ideally, breeders will select individual plants with the same grain yield as the commercial hybrids to conserve the desired tolerance by using marker-assisted selection tools.

Selection for early and late cold tolerance under field conditions is often the most affordable way to select cold-tolerant genotypes, which can germinate, grow, and develop under challenging circumstances where sensitive genotypes cannot. In this sense, several sorghum hybrids have been developed under traditional breeding methods. These hybrids show a good adaptation to climatic conditions, where no other sorghum can grow and produce flowers, pollen, and seeds successfully (Osuna-Ortega et al., 2003; Leon-Velasco et al., 2009). However, variable climatic conditions might make a difficult selection for cold temperature stress. Furthermore, the multigenic nature of cold tolerance makes the evaluation difficult because of the genotype–environment interactions in the field. Controlled conditions are recommended for further research, which involves greenhouse screening for cold tolerance in combination with field evaluation in multi-environment conditions (Kapanigowda et al., 2013). For breeding programs, the application of short and intense chilling is more promising (Windpassinger et al., 2017). Germination tests at low temperatures have been proposed as a selection tool for early establishment in sorghum (Singh, 1985; Brar and Stewart, 1994), but there is a poor relationship association between germination in the laboratory and field selection for cold seedling tolerance because of its low heritability or probably low repeatability (Emendack et al., 2021). The flowering and maturity delay is observed when sorghum is subjected to cold temperatures after emergence (Kapanigowda et al., 2013; Maulana and Tesso, 2013), although a significant reduction of grain yield is not found (Maulana and Tesso, 2013; Windpassinger et al., 2017). A strong expression of heterosis (vigor hybrid) is desirable for yield and chilling tolerance in sorghum (Windpassinger et al., 2017). Sorghums with different degrees of cold tolerance represent an opportunity to know and integrate the physiological, metabolic, and molecular mechanisms associated with chilling stress tolerance and can help to identify the complex networks involved in plant tolerance to cold stress.

## Future research for a comprehensive understanding of sorghum cold stress response

We have integrated the advances in the knowledge of sorghum in response to cold stress, describing the physiological, biochemical, metabolic, and molecular changes in response to cold stress in sorghum. This will improve the understanding and integration of the models involved, such as the ICE-CBF-COR model conserved in several plants.

The broadening and deepening of knowledge using different tools for massive sequencing of miRNAs, mRNAs, metabolomics, and proteomics will be key pieces that will help to better integrate the response effect of cold stress in sorghum. In this sense, the best networks could be proposed as good candidates for functional analysis for subsequent use in genetic engineering using tools such as the CRISPR-Cas system. Validation of the miRNA–target gene–protein networks will help to provide novel insights into the contribution to cold tolerance in sorghum and the genetic mechanisms involved, which will also help to develop stress tolerant plants. Upcoming efforts in the search for sorghum genes that are potentially able to confer cold tolerance will undoubtedly be more successful if high-throughput techniques, such as microarrays, next-generation sequencing, and proteomics, are more extensively used.

An essential part of the understanding of cold stress response in plants is influenced by epigenetics. Therefore, it will be relevant to consider sorghum varieties with different degrees of cold tolerance and multi-environment conditions for cold response. Moreover, it is important to consider that the rhizosphere microbiomes retain their potential to promote plant growth at low temperatures is a worldwide trend in the field of agricultural inoculation technology (Malusá et al., 2012; Pandey and Yarzábal, 2018). Therefore, the sorghum genotype–environment–rhizosphere microbiome interaction will be essential for selective breeding of sorghum for cold stress tolerance. This will provide an opportunity to study, understand, and integrate the mechanisms associated with cold stress. In the long term, the knowledge generated by breeders and plant biologists will help to understand the intricate molecular mechanisms governing stress response under challenging situations to develop more cold temperature–tolerant plants, allowing higher yields under difficult conditions to support profitable crops to feed the world’s growing population.

## Conclusion

Comprehensive understanding of the physiological, biochemical, and molecular response mechanisms to low temperatures in sorghum can contribute to the selection of cold-tolerant genotypes and crop improvement. Here, we reviewed and discussed some of the research results that focused on these mechanisms and signal transductions and the molecules involved in sorghum cold stress, which will be essential information for future studies. Much advancement has been achieved in understanding cold tolerance in sorghum. Although there are interesting proposals for the function of some lipids, sugars, nitrogen compounds, TFs, and proteins in sorghum cold stress, it is crucial to elucidate the interactions between these elements, which could be essential to clarify the sorghum cold stress response. Still, reduced yield caused by low temperatures remains a problem, especially in high altitudes where sorghum is cultivated. In addition, we should consider that the incidence of extreme temperatures is expected to increase as part of general global warming, and losses caused by cold temperatures may increase (Raza et al., 2019). Cold-tolerant sorghum varieties will need to be bred, allowing the cultivar to tolerate, survive, and develop when exposed to cold. A strategy that considers breeding and genetic engineering tools confers cold tolerance in sorghum plants.

## Author contributions

PH and FR contributed to the conception of the manuscript and drafted the manuscript. PH, LM and FR contributed to manuscript preparation and approved the final manuscript. All authors contributed to the article and approved the submitted version.
